# A Modified Three-Dimensional Negative-Poisson-Ratio Metal Metamaterial Lattice Structure

**DOI:** 10.3390/ma15113752

**Published:** 2022-05-24

**Authors:** Fangyi Li, Qiang Zhang, Huimin Shi, Zheng Liu

**Affiliations:** 1 School of Mechanical and Electrical Engineering, Guangzhou University, Guangzhou 510006, China; gdzq7377@163.com (Q.Z.); hmshi@nwnu.edu.cn (H.S.); 2 College of Physics and Electronic Engineering, Northwest Normal University, Lanzhou 730070, China

**Keywords:** metamaterials, negative-Poisson-ratio, lattice structure, numerical simulation

## Abstract

Mechanical metamaterials are of interest to researchers because of their unique mechanical properties, including a negative Poisson structure. Here, we study a three-dimensional (3D) negative-Poisson-ratio (NPR) metal metamaterial lattice structure by adding a star structure to the traditional 3D concave structure, thus designing three different angles with a modified NPR structure and control structure. We further study the mechanical properties via finite element numerical simulations and show that the stability and stiffness of the modified structures are improved relative to the control structure; the stability decreases with increasing star body angle. The star angle has the best relative energy absorption effect at 70.9°. The experimental model is made by selective laser melting (SLM) technology (3D printing), and the compression experiment verification used an MTS universal compressor. The experimental results are consistent with the changing trend in finite element simulation.

## 1. Introduction

In the past few decades, lightweight lattice materials have received widespread attention as engineering materials because they have excellent properties that natural materials do not have. Lattice structures have light weight, low density, high strength, and strong specific energy absorption [1,2,3,4,5,6,7,8,9,10,11]. They have widely been used in vehicles, ships, aerospace, marine engineering [12,13], etc. Their fine microstructure design has been used to create some unconventional mechanical properties, such as a negative-Poisson-ratio (NPR), negative compressibility, and negative stiffness. Thus, lattice materials with NPR properties have widely been used in engineering because of their excellent fracture resistance [14,15,16,17], indentation resistance [18,19], sound absorption [20], and impact resistance [21].

The pioneering work of Lakes [15], Caddock, and Evans sparked interest in NPR materials. An increasing number of NPR structures are being discovered, manufactured, and synthesized. In recent years, two-dimensional (2D) metamaterials with simple production and easy analysis have become popular [22,23,24]. Designs include a concave structure [25], star structure [26,27,28,29,30], chiral structure [31,32], hexagonal honeycomb structure [33], digging structure [17], and reticulated structure [27]. The 2D honeycomb structure proposed by Gibson is one of the most textbook auxiliary metamaterials.

As 2D metamaterials cannot meet all needs, three-dimensional (3D) metamaterials with better performance are of interest [34,35,36,37,38,39,40,41]. For example, NPR tubular structures [17,42,43], tension–torsion coupling structures [44,45], double-arrow energy-absorbing structures [46,47], torsional structures [44,48,49], and 3D hexagonal reentrant structures [50,51,52,53] stand out. Of the 3D re-entrant structures, Li et al. [54] proposed a new 3D NPR concave lattice based on a 2D NPR structure summarized by predecessors. They performed physical experimental verification and proposed an enhanced version of the 3D NPR concave lattice with pillars. They adjusted the performance of the NPR structures by adjusting the pillars and then compared the compressive and bending resistance of the 3D concave lattice. The results were mediocre.

More recently, Li et al. [55,56] considered the direction of energy and studied a 3D concave lattice using the energy method, which is still used by scholars as a reference for such research. Subsequently, Xue et al. [57] studied compression performance by designing four 3D concave lattices and concluded that the structural compression performance was proportional to the NPR performance of the unit. Shen et al. [58] rotated a single concave beam structure by 90 degrees on the basis of the classical 3D concave honeycomb lattice. They then connected four connecting ribs at the concave midpoint and used experiments and numerical simulations of the four models to show that the mechanical properties and energy absorption capacity of the structure could be effectively improved. Existing NPR structures are mostly 2D or 3D due to manufacturing difficulties and many other factors. They are based on plastics and composite materials, and there is little research on 3D metal structures with an NPR. Structural experiments on NPRs for 3D metal materials are also lacking.

Therefore, in this paper, a modified 3D re-entrant NPR metamaterial metal lattice structure, along with a combination method, is proposed. We verified the material through numerical simulations and experiments. The stability and mechanical properties of the 3D honeycomb structure lattice were changed by adjusting the angle of the 3D concave structure and the lattice of the star structure by combining the concept of an NPR with a metal lattice structure. Three different angles of the 3D honeycomb structure and control structure were designed, and a numerical simulation analysis was performed. One of the models was made via SLM laser 3D printing technology for experimental verification, and the results showed that the modified structure can nicely improve stability and energy absorption capacity. A larger angle of the star structure (∅) implied worse stability of the honeycomb structure.

## 2. Design and Manufacture of a Modified 3D NPR Structure

### 2.1. Modified 3D NPR Structural Design

**Figure 1:**
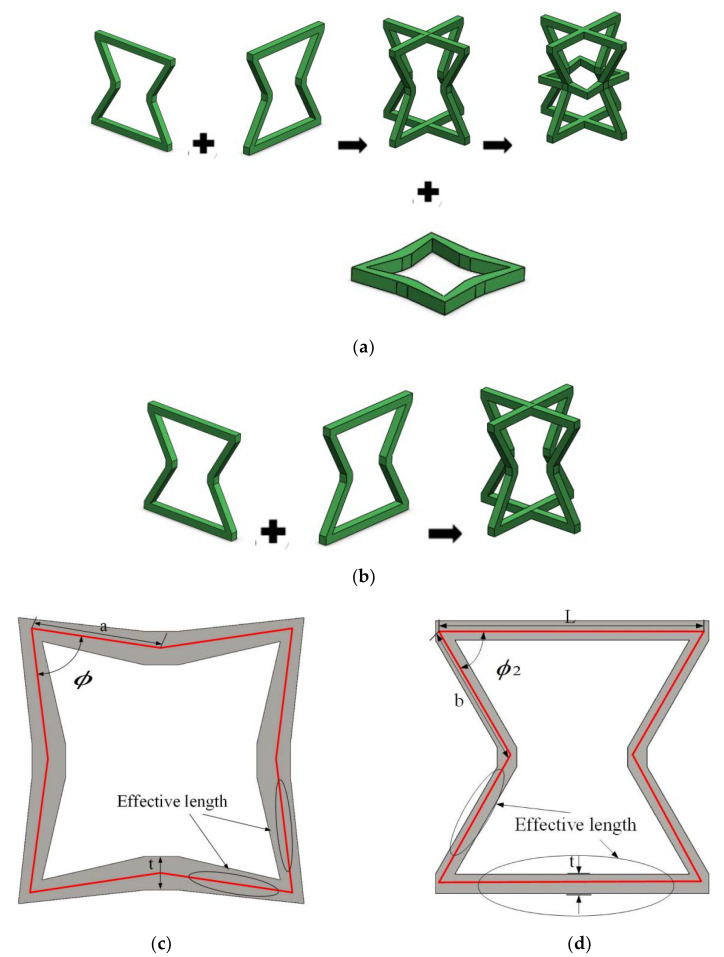
Modified NPR 2D and 3D structure configurations. (**a**) Modified 3D NPR structure configuration; (**b**) Schematic diagram of the 3D component unit structure of the control model; (**c**) Geometric structure configuration of 2D star structure components; (**d**) geometric structure configuration of 2D concave structure components.

A modified 3D NPR lattice structure with the advantages of a concave structure and a star structure was designed. Its cell structure is shown in Figure 1a, and the two concave structures are placed together. One of the concave structures rotates 90° in the direction of the straight edge midline to form the basic framework. The stellate section is perpendicular to the straight midline of the concave structure, and its four concaves are placed in conjunction with the four concaves of the basic framework; the two concave structures and one stellate are orthogonal and fixed in the concave direction to form a 3D structure.

To highlight the advantages of the modified 3D NPR structure, three corresponding control models were also designed. These are similar except for the absence of star-shaped structures, which are consistent with the corresponding structures; the cytomembric composition is shown in Figure 1b. All lengths of the eight bevel arms of the two-dimensional star structure in Figure 1c are indicated as “a”, and the four angular sizes are all ∅. Figure 1d shows that the bottom edge length of the 2D concave structure is L, the length of the four hypotenuses is “b”, and the angle between the bottom edge and the hypotenuse is ∅_2_. The values of the three model plane parameters are shown in Table 1.

**Figure 2:**
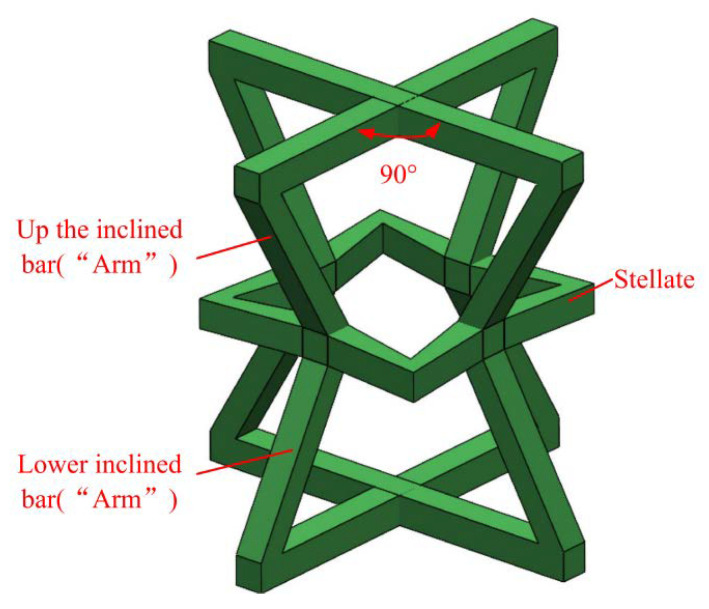
Schematic diagram of structural single cells.

**Figure 3:**
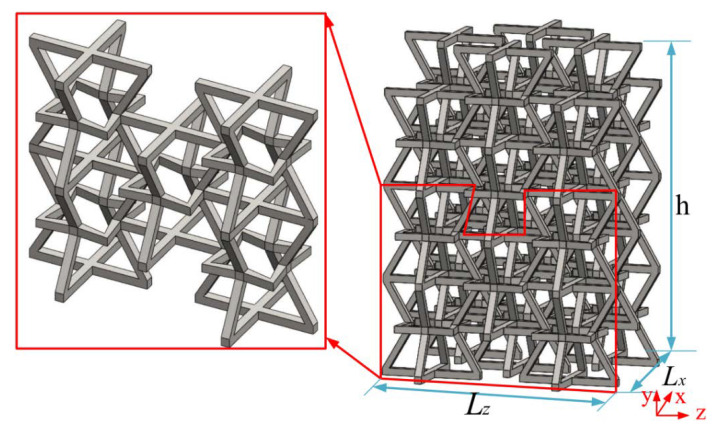
Modified negative-Poisson-ratio structure composition.

**Figure 4:**
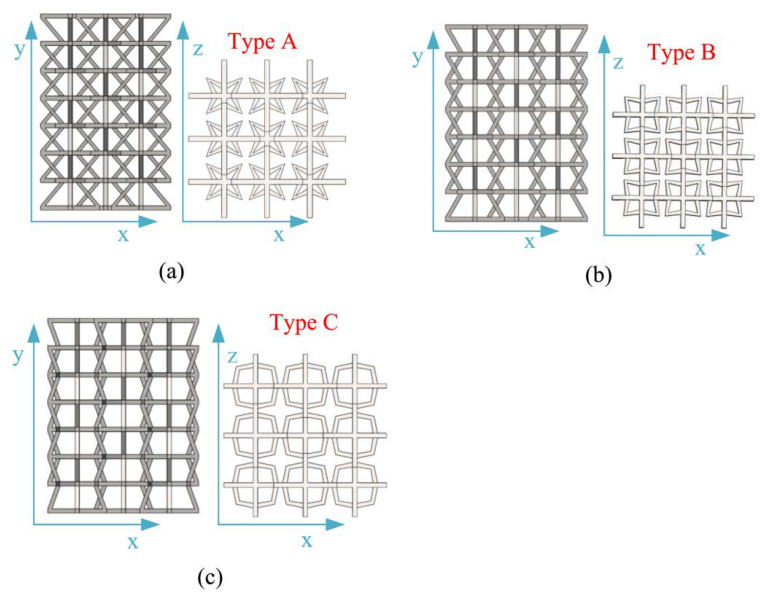
Different 2D plane compositions: (**a**) type A, face-up and overhead composition; (**b**) type B, face-up and overhead composition; (**c**) type C, face-up and overhead composition.

Figure 2 shows a schematic diagram of the structure, which consists of four upper inclined rods with four lower oblique rods and stellate rods. One upper inclined rod of a 3D structure overlapped with a 3D structure and one lower inclined rod. The lower inclined rod overlapped with the upper inclined rod of another 3D structure. According to the above combination method, the honeycomb combination structure (Figure 3) is formed by further repeated arrangement via spatial direction expansion. The height of the 3D honeycomb structure is h in the Y direction, length is L_x_ in the X direction, width is the length in the Z direction is L_z_, and thickness is in “t”. In this paper, the mechanical properties of the negative-Poisson-specific metamaterial were studied by taking the three types of honeycomb types (A, B, and C of 3 × 3 × 3), i.e., ∅_2_ = 50°, ∅_2_ = 60°, and ∅_2_ = 70°. The 2D plane composition is shown in Figure 4, and the main parameters of the honeycomb structure of the three types are shown in Table 2.

### 2.2. Manufacture of a Modified 3D NPR Structure

**Figure 5:**
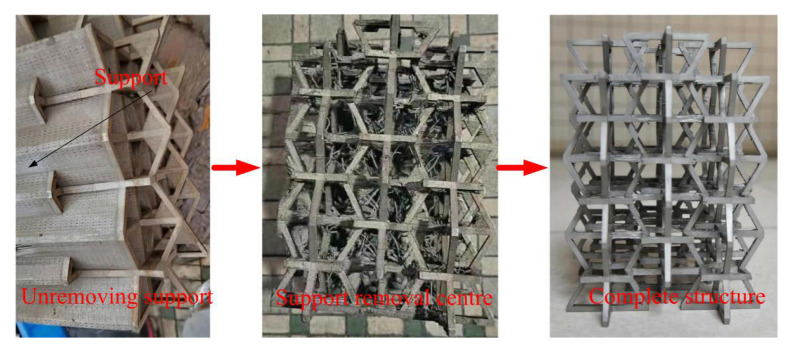
The 316 L NPR structural support-removal process.

The experimental model in this article was made using a Hanbang laser SLM-280 additive manufacturing 3D printer for 316 L stainless steel honeycomb structure printing, with a working laser power of 250 W, and a layer thickness of 50 μm, and produced at a melting temperature of 800 degrees. First, SOLIDWORKS 3D modeling software was used to model and export the STL file. The 316 L metal powder or fine particles were melted using a 3D printer with a high-energy laser to make it into the required 3D shape of the slice. The sintering machine then accumulated these slices layer by layer to obtain the required parts. The SLM process generally needs to add a support structure due to the difficulty of the support-removal process, thus resulting in insufficient accuracy. There is a need for post-reprocessing to improve accuracy, which is a shortcoming of the SLM laser 3D printing technology. The structure support removal process is shown in Figure 5.

## 3. Modified 3D Negative-Poisson-Specific Lattice Test

**Figure 6:**
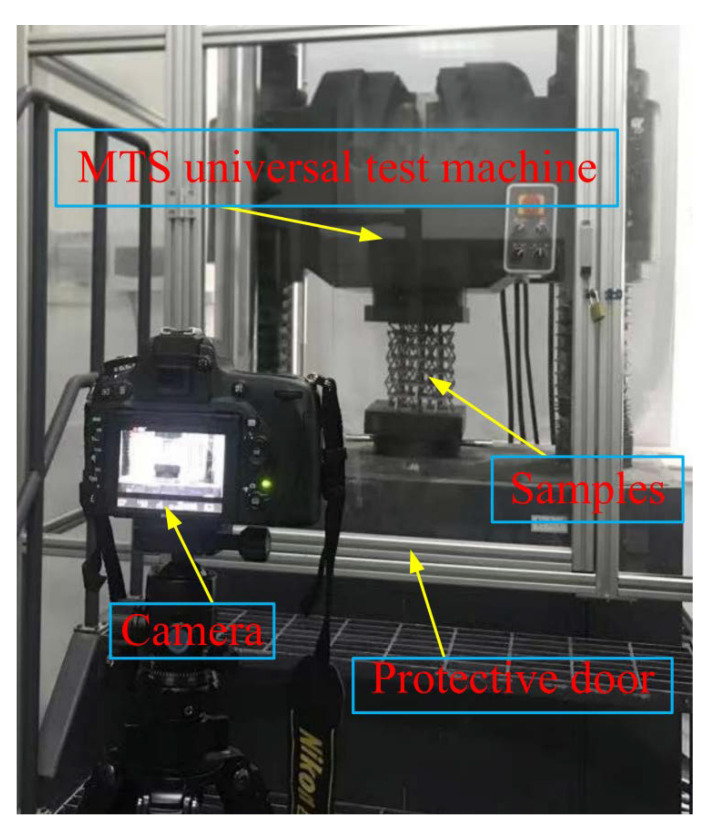
MTS universal testing machine.

**Figure 7:**
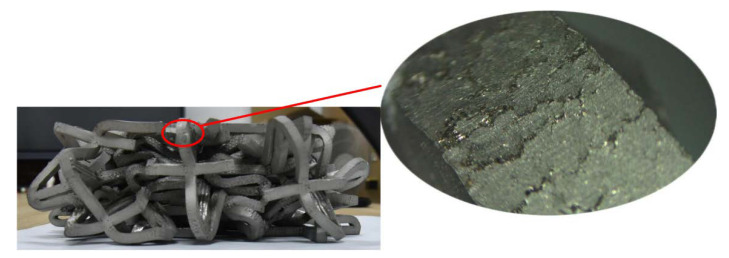
Local failure of lattice cracks.

In this experiment, an MTS universal testing machine was used for quasi-static compression experiments, compressing 180 mm at a destructive speed of 5 mm/min (Figure 6). An MTS universal testing machine with a safety gate was adopted to make experimentation safe and effective. The lower clamp was a fixture with a “ball kettle”, which would be tilted according to the change in force. This ensured that the experimental sample would not “fly” out of the test bench after being forced. Figure 7 shows the local failure cracks of the lattice after the experiment. It clearly shows that the failure cracks are relatively “regular” and striped. At the same time, it can be seen that the surface is also relatively rough. Specifically, at the junction of the two surfaces, the uneven texture is due to the traces left by surface treatment after the production was completed.

## 4. Finite Element Numerical Simulation Analysis

### 4.1. Performance of 316 L Stainless Steel

**Figure 8:**
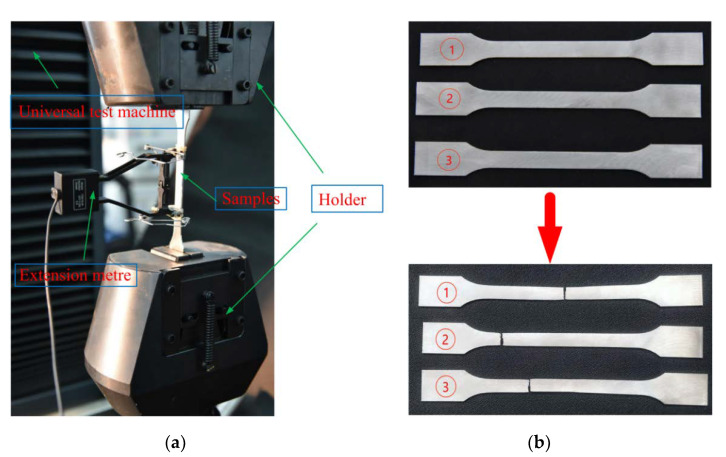
Single-axis tensile test of 316 L sample: (**a**) test device; (**b**) samples.

**Figure 9:**
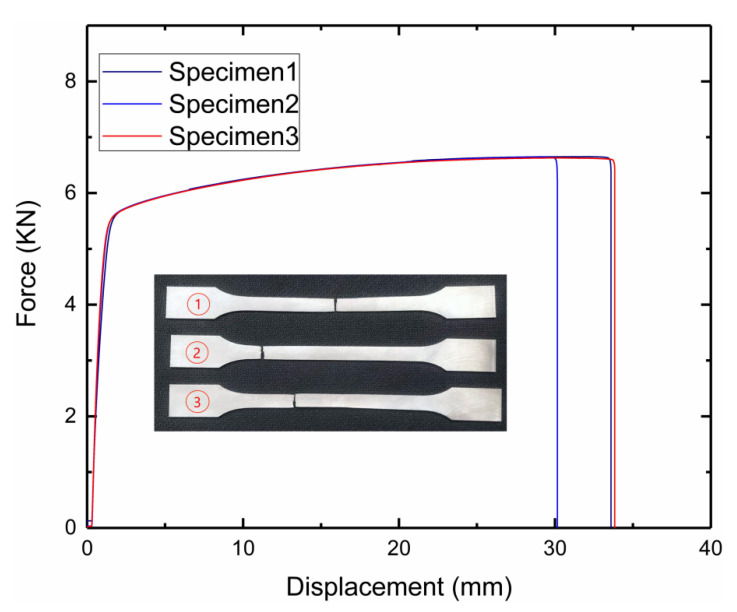
Uniaxial tensile test’s load–displacement curve.

A LABSANS-LD26.504 universal material testing machine (Figure 8a) was used for uniaxial tensile experiments. During this process, the performance parameters of the 316 L stainless steel material were evaluated. The initial distance of the extensometer was 5 mm, and the loading speed was 2 mm/min for the sample uniaxial tensile test. The three samples used for testing were all in accordance with the GBT228-2002 tensile specimen national standard, which recommended a thickness of 1 mm, using a Hanbang laser SLM-280 additive manufacturing 3D printer. The three samples before and after the destruction are shown in Figure 8b (1,2,3), and the main experimental parameters are shown in Table 3. The table indicates that the mechanical properties of stainless steel prepared by SLM 316 L are different from those of ordinary 316 L stainless steel; the elastic modulus, tensile strength, density, and Poisson’s ratio performance are close, but the yield limit of ordinary 316 L stainless steel is much smaller than 316 L prepared via SLM because the SLM process forms austenite at a high temperature of 800 °C while preparing 316 L stainless steel. This, in turn, improves yield strength and toughness. The measured failure load–displacement curve is shown in Figure 9.

### 4.2. Finite Element Model Establishment

SOLIDWORKS software was used for 3D structure modeling, and three models of ∅_2_ were established as 50°, 60°, and 70° (type A; type B; type C) and the corresponding three control models (control A; control B; control C). These were saved in IGES file format, imported into Abaqus CAE commercial software, and quasi-static compression simulation experiments were performed to study the mechanical properties at different angles.

The measured experimental parameters were entered into the Abaqus CAE Material Manager. The model was set to beam elements, and the material properties were assigned to the finite element model.

**Figure 10:**
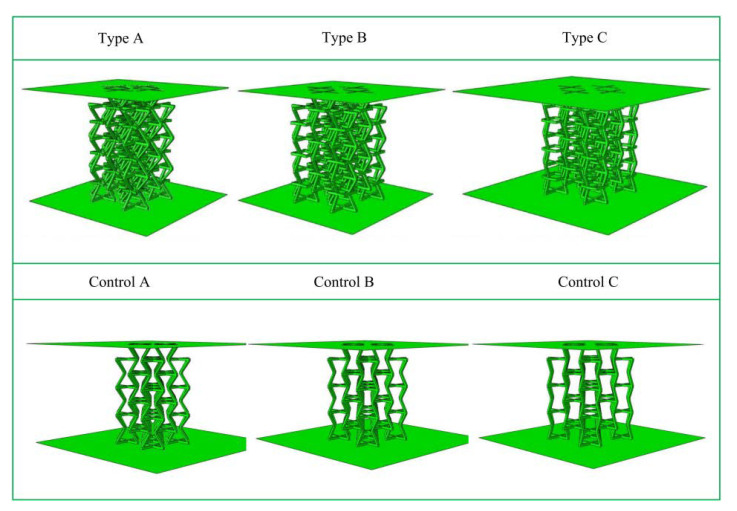
Schematic diagrams of six models for numerical simulation.

**Figure 11:**
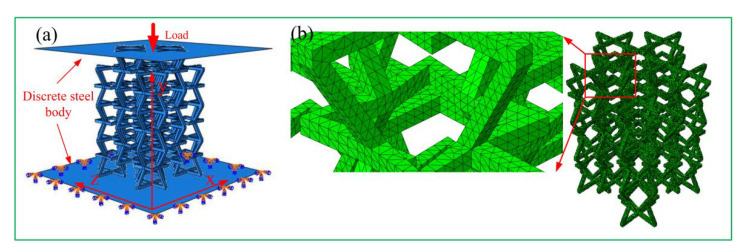
Establishment of a finite element model: (**a**) payload application; (**b**) grid division.

Figure 10 shows that the two plates above and below the structure were assembled with discrete rigid bodies. The finite element model of the entire structure was defined as self-contact. Surface-to-surface contact was adopted between the upper and lower steel plates. The penalty contact method was used, and the friction coefficient was 0.2. As shown in Figure 11, the lower steel plate was completely fixed, and a displacement load of 180 mm in the negative Y direction was applied to the upper steel plate. The analysis step was calculated and analyzed using display dynamics, and the calculation time of the model was set to 1 s. As shown in Figure 11b, a manual partition was used for seven layers in order to obtain a higher mesh quality. Each layer of the star structure was then divided separately, and the global seed distance of the concave structure was 3.2. The global seed distance of the star structure was 3.2, the curvature control and the minimum size control were 0.1, and the mesh attribute was a tetrahedral free technical division, with 106,698 tetrahedral mesh elements; the amplitude was calculated using amplitude-smoothing steps.

### 4.3. Analysis and Discussion of Mechanical Responses of Finite Element Models

**Figure 12:**
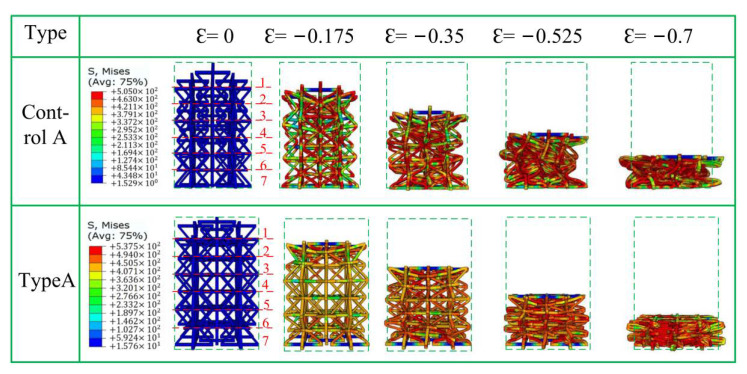
Fragmentation process of type A in the y direction.

**Figure 13:**
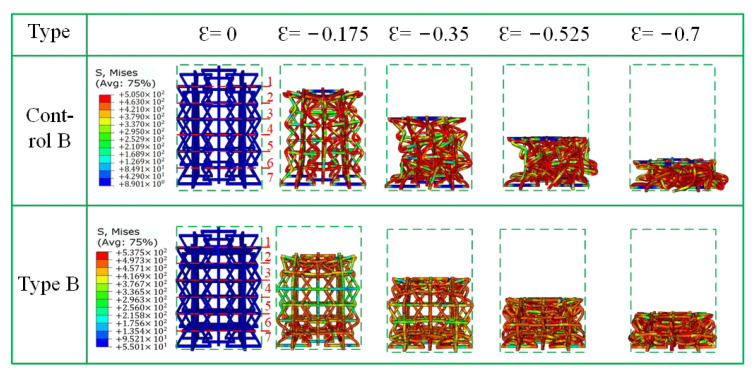
Fragmentation process of type B in the y direction.

**Figure 14:**
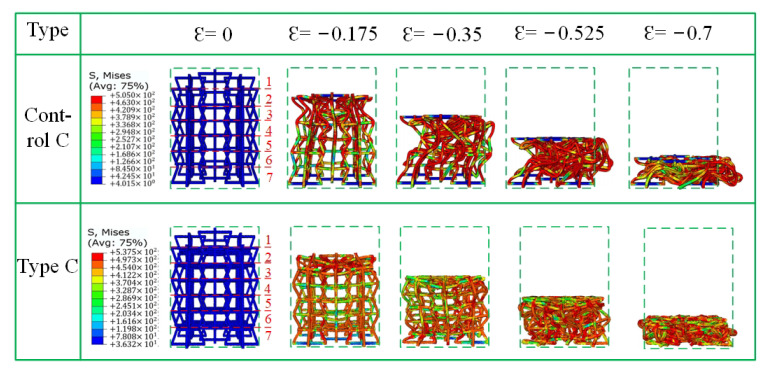
Fragmentation process of type C in the y direction.

Figure 12, Figure 13 and Figure 14 show the crushing patterns of control A, type A, control B, Type B, control C, and type C, respectively, in the y direction under different strains. The simulation results show that the stability of the modified honeycomb lattice with the addition of the star structure is much better than that of the control structure without the star. In this paper, the strain is considered negative when the structure is compressed, and positive when the structure is compressed. Figure 12 shows that control structure deformation mainly begins from the first layer when the ε = −0.175. In Figure 12, Figure 13 and Figure 14, Arabic numerals 1 to 7 at ε = 0 represent the first-to-seventh layers, respectively. The structure then begins to gradually deform downwards. The deformations of the second-to-sixth layers are more uniform, and there is an NPR phenomenon of inward re-entrant.

Type A materials are observed under the same strain. The deformation trend of the first layer is similar to the control structure. The difference is that the force deformations of the second and sixth layers are relative to the deformations of the third-to-fifth layers. The force situation is more concentrated in the center of the second layer and the sixth layer because of the structural design: the “arms” connected at both ends are relatively smaller than the number of intermediate layers.

At ε = −0.35, control A appears slightly to the left convex phenomenon under the action of displacement load. The single-cell structure appears to undergo varying degrees of deformation, and the single-cell “arm” is unevenly forced. The honeycomb structure is “distorted”. The figure shows that the first layer is completely pressed into the second layer, and the fifth layer of shape variables is second only to the first layer. The first and last ends of the structure still show an inward re-entrant phenomenon. Type A has a star-shaped structure, and there is no convexity in control A under the same displacement load; the stability is improved.

At ε = −0.525, control A shows obvious macroscopic convexity. There is no re-entrant phenomenon at ε = −0.175. Type A is already in the dense stage, but upon “embedding” in the first layer, the second and third layers do not show the usual “expansion” phenomenon. Rather, they show an abnormal concave phenomenon that is quite obvious. This is likely the cell “oblique arm” in the case of large deformation that pulls the cell horizontal “arm”, thus leading to inward re-entrant.

Type B and control B in Figure 13 show nearly the same phenomenon as that observed in type A and control A in Figure 12, except that the NPR phenomenon of control B at ε = −0.175 is relatively more obvious than that of control A. A “dumbbell”-shaped phenomenon appears on the macroscopic level. In type B, there is a slight “dumbbell”-shaped phenomenon at ε = −0.525.

Type C shows a different crushing situation than the first two types (Figure 14) when ε = −0.35. There is a “distortion” phenomenon toward “convexity” because one side of the sixth layer is loaded in the previous period due to the influence of the star angle. There is a tilt effect that results in a concave arm of the sixth layer being deformed from side to side, thus resulting in distortion, which further leads to subsequent unstable offsets.

In summary, the control structure without a star has a more obvious macroscopic negative Poisson phenomenon of the honeycomb structure when the strain is small, and when the increase in the angle of the concave structure is $\emptyset_{2}$. The modified structural stability of the stellar body is better than the structural stability of the non-star body, but the NPR effect is worse. This is due to the reduction in the stress generated by the deformation of the star, compared with the stress generated when the concave body is “concave.” In addition, the ∅ angle of the stellar body is inversely proportional to the stability of the star honeycomb structure.

**Figure 15:**
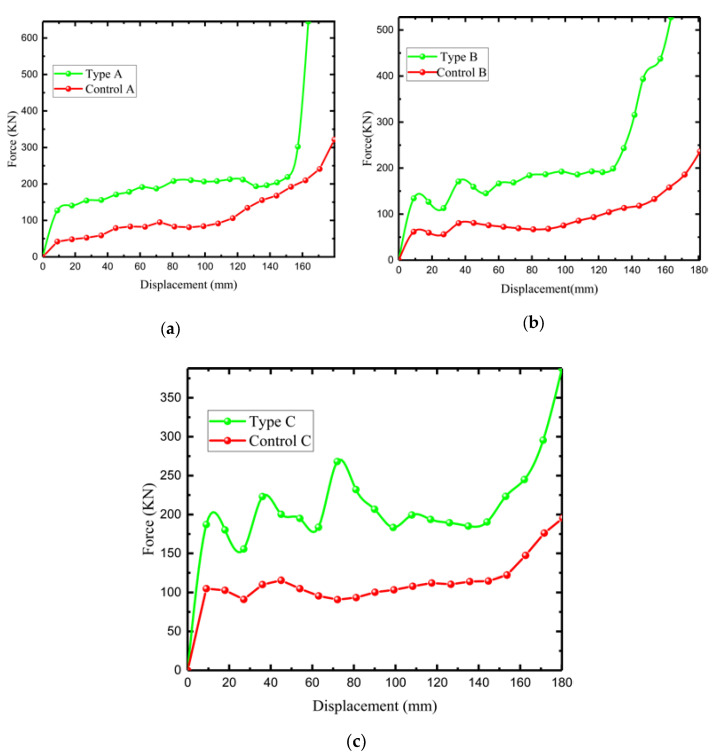
Three types of load–displacement curves. (**a**) Comparative load–displacement curve of type A; (**b**) Comparative load–displacement curve of type B; (**c**) Comparative load–displacement curve of type C.

Figure 15 shows the load–displacement relationship between the three novel lattices and the control lattice. The finite element results of Abaqus CAE show that the load performance of the modified negative-Poisson-specific lattice is greater than that of the control group, which is due to the addition of star structure to the modified negative Poisson rather than the honeycomb lattice. This makes the deformation of each layer of the lattice more uniform under loading conditions. The stability and stiffness of the structure are improved. The modified lattice curve has obvious fluctuations. There is an increase in the angle ∅ of the modified structure. There is a greater fluctuation amplitude because the modified structure can be stabilized and destroyed from end to end until the second-to-fifth layers begin to contact each other and are relatively uniformly destroyed. The relative fluctuation of the control lattice is smaller, but the fluctuation also increases with the increase in the angle of the concave structure $\emptyset_{2}$ (but only with a small ε).

We studied the angle of the concave structure $\emptyset_{2}$ with increasing ε: The curve fluctuates in the plain stage because the control structure does not occur when the ε is small after load deformation. Furthermore, the “convex” phenomenon does not occur when ε increases. There is a more obvious, macroscopic “convexity” phenomenon in the structure, which shows that the control lattice is larger and more stable with increasing $\emptyset_{2}$.

**Figure 16:**
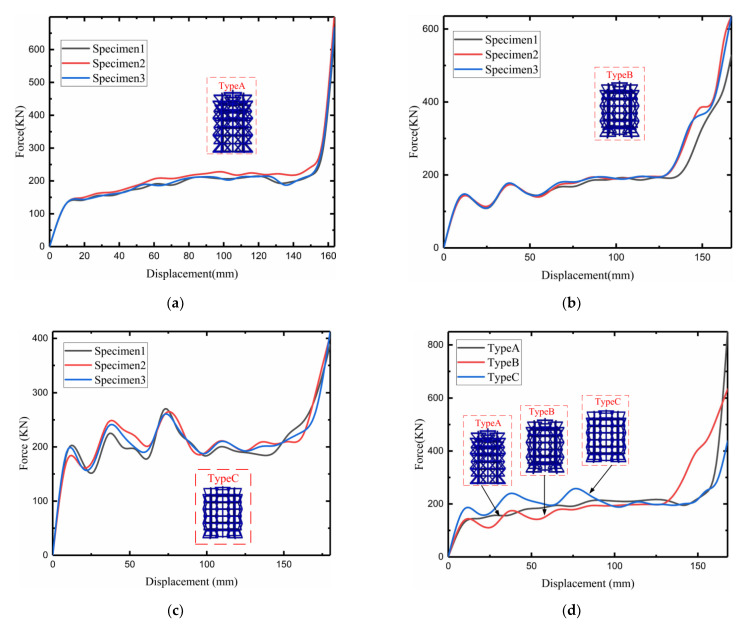
Load–displacement curve of numerical analogue: (**a**) type A load–displacement curve; (**b**) type B load–displacement curve; (**c**) type C load–displacement curve; (**d**) three types of fitting simulation load–displacement curves.

Figure 16a–c show the load–displacement variations simulated by the modified negative-Poisson-specific lattice under the mechanical parameters of three samples. The simulation results show that the three modified 3D NPR compression load–displacement curves change similarly and can be roughly divided into the initial stage, the plain stage, and the dense stage. The initial stage of the force rises sharply until the first peak force is reached. The first peak forces of types A, B, and C are about 150 (KN),150 (KN), and 200 (KN). At this time, the lattice is destroyed, transitioning from the elastic deformation stage to the plastic deformation stage. Entering the plain stage, the displacement increases sharply, but the force changes almost slightly; specifically, in type A, the peak and trough difference is controlled within 10 (KN), and the maximum difference between the peak and the trough of type B reaches 30 (KN), which is because of entering the plain stage; thus, structural buckling is reduced. However, peak and trough difference in type C is approximately 100 (KN), because when the size is larger, the structure does not buckle more easily.

Buckling after the first peak force also increases with increasing ∅ and $\emptyset_{2}$. This is because the increase in ∅ and $\emptyset_{2}$ after the structure is loaded causes the degree of failure of the lattice to propagate layer by layer. After reaching the densification stage, the relationship between the load and crushing displacement increases exponentially due to the increasingly smaller pores between the first and seventh layers of the structure; thus, the re-displacement change results from an exponential increase in the previous load. Figure 16d shows the load–displacement change, simulated after fitting by the mechanical parameters of the three samples. The peak force of type B is better than that of the other two sets of models, which is sufficient to indicate the superiority of the type B structure.

### 4.4. Finite Element Poisson’s Ratio Analysis and Discussion

**Figure 17:**
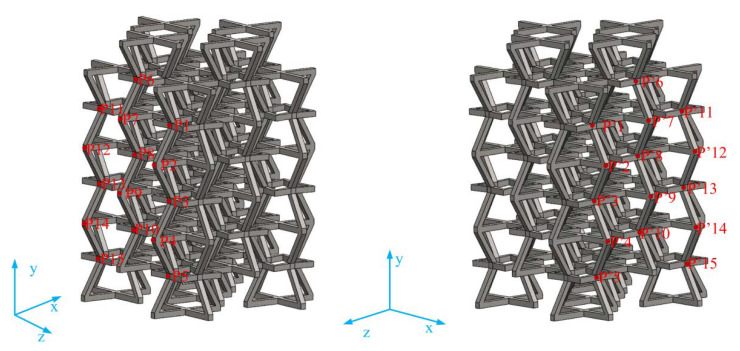
Schematic diagram of the lateral strain points of the simulated structure negative-Poisson-specific space.

**Figure 18:**
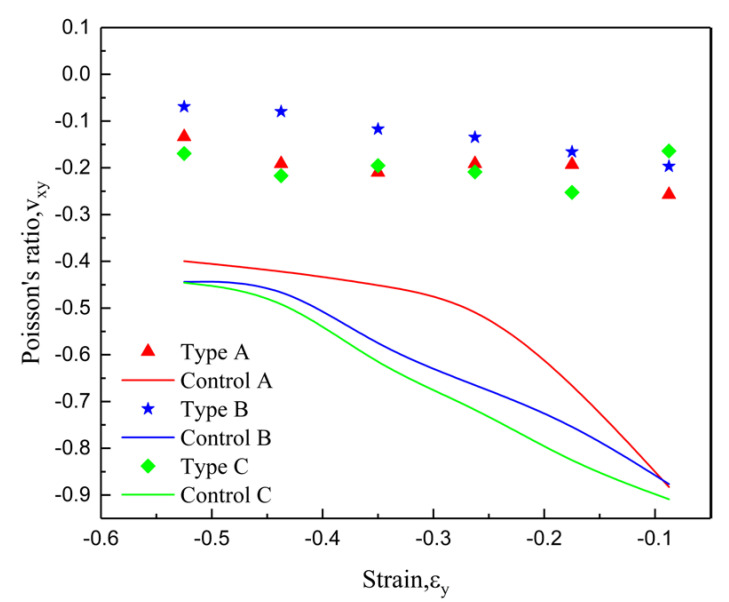
Simulation Poisson’s ratio comparison: Poisson’s ratio–strain curve comparison for type A, type B, and type C, as well as control A, control B, and control C.

In order to measure the Poisson ratio of the structure, the lateral strain of the structure was calculated by taking 15 spatial points shown in Figure 17 (x, y, and z represent the coordinate axes.), and the Poisson ratio calculation results are shown in Figure 18. The results show that the lateral deformation of the improved structure is much smaller than that of the control structure. As can be derived from Figure 18, the minimum value of the Poisson ratio is about −0.25, and the maximum value is above −0.85, which is caused by the addition of the star structure that makes deformation less difficult. Interestingly, the negative-Poisson-ratio effects of control A, control B, and control C sequentially increase, all of which occur due to an increase in the angle of the concave, which results in an increase in transverse strain. However, the improvements in types A, B, and C do not show this phenomenon, which is due to the influence of the star body angle ∅, making the negative Poisson-ratio-effect of type B secondary to that of types A and B.

### 4.5. Energy Absorption

The specific energy absorption calculation adopts the total mass of the structure on the load–displacement curve integration ratio. The energy efficiency ESA, $E_{s}^{c}$, and the theoretical calculation formula is proposed by Li [59] et al. as follows:

$$E_{s}^{c}=\frac{\int_{0}^{\Delta_{x}}P\left(x\right)d\left(x\right)}{m_{g}},$$

where $\Delta_{x}$ is the crushing distance, P(x) is the load–displacement curve equation, and ${\;m}_{g}$ is the structural mass, respectively.

**Figure 19:**
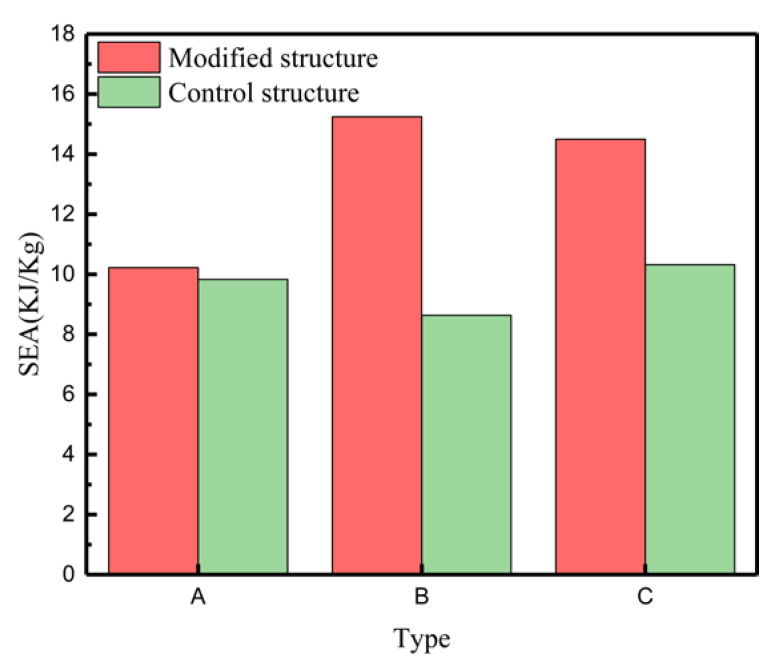
Specific energy absorption (SEA) under modified and control structures.

The specific energy absorption case is calculated by theoretical calculation [60] similar to that obtained by the finite element calculation in this paper (Figure 19). The specific energy absorption (SEA, energy absorption per unit mass) of the modified structure and the control structure is shown in Figure 19. Here, the abscissa represents the type (i.e., A represents type A and control A; B represents type B and control B; C stands for type C and control C). It can be clearly seen from Figure 19 that type B has the best energy absorption effect, with a specific energy absorption of 16 (KJ/Kg), while type C is slightly inferior, with a SEA value of about 15 (KJ/Kg), and type A has the worst effect in the new, modified structure.

The modified structure can also effectively improve the energy absorption effect relative to the control group structure. When compared with the traditional structure, type B is the best, as the change in the level of increase in SEA is nearly 7 (KJ/Kg). This is because the addition of the star shape results in improved rigidity of the structure, as well as the improved capacity of energy absorption. Clearly, the star body angle ∅ has the best energy absorption effect, at 70.9°. The performance effect decreases as the ∅ increases or decreases.

## 5. Finite Element Model Comparison between Experiments

**Figure 20:**
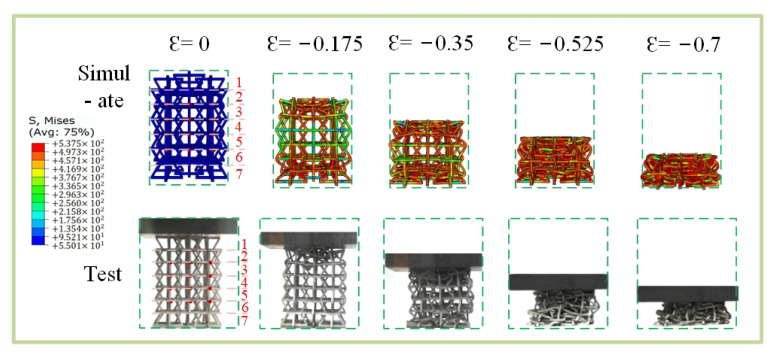
Comparison of simulation experiments in the x direction.

Figure 20 shows that the simulation differs slightly from the experiment with macroscopic crushing under uniaxial compression. In Figure 20, Arabic numerals 1 to 7 at ε = 0 represent the first-to-seventh layers, respectively.The first-layer change is basically the same at ε = 0.175. Changes in the sixth and seventh layers are also highly consistent when ε = 0.35. However, the difference is that the test model has a “convex” phenomenon during the crushing process, which occurs because the clamp under the MTS universal testing machine used has a “kettle-ball” design. The initial placement is uneven, or the force imbalance causes the lower clamp to tilt, thus resulting in the tilt of the entire test model. In addition, the 3D printing technique used to make models can damage accuracy, especially while removing supports; thus, the deformation of the experimental model is uneven after loading. The ball kettle tilts, but the deformation mode of the experimental model is highly consistent with the deformation mode of the simulation.

**Figure 21:**
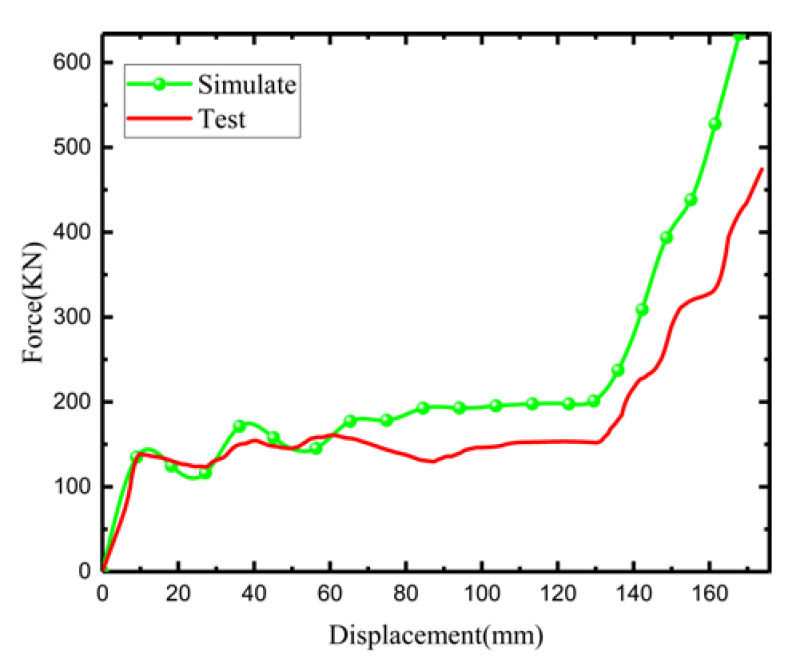
Comparison of load–displacement curves in simulation and experiment (type B).

Figure 21 shows a load–displacement curve plot under the uniaxial compression experiment of the experimental model and the simulation model. All show the initial stage, the plain stage, and the compact stage. The initial phase and first peak force show highly consistent trends, both around 150 (KN), while peak forces in the plain phase and densification phase are smaller than those in the simulation model. In the plain stage, the minimum load difference is almost 0 (KN), while the maximum load difference reaches 30 (KN), which is due to the error resulting from the convexity of the model during the experiment. The removal of support during the production process results in the same crushing displacement. Thus, the load is smaller than that in the simulation model. The trend is highly consistent because of the convexity of the model in the experimental process.

In order to measure the Poisson’s ratio of the experimental specimen, a camera was used to detect the transverse strain of 16 points in the sample in Figure 20, and then the Poisson’s ratio was calculated via image processing. The 16 points calculated with Poisson’s ratio of the simulated specimen were taken in different directions from Figure 17, but the calculation results are highly consistent, and the error range is around −0.01.

**Figure 22:**
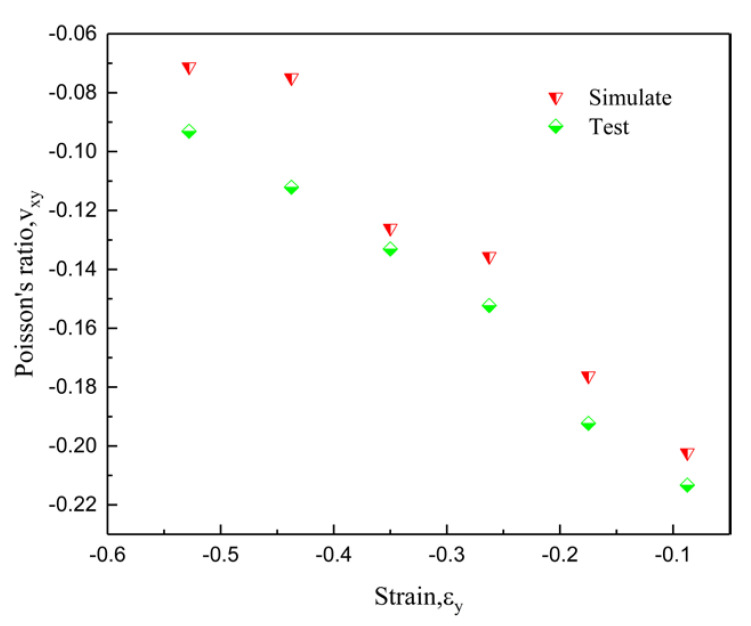
The comparison of Poisson’s ratio–strain curves for experiment and simulation.

Poisson’s ratio–strain curves in Figure 22 show that the experiment and simulation results are roughly similar. The Poisson ratio is the smallest at ε = −0.375, and the difference is only about −0.01. When ε is less than 0.375, the Poisson ratio differs relatively widely, reaching around −0.04. The reason for this phenomenon is that, during the production of experimental samples, especially when the support is removed by hand, the thickness of some arms is inconsistent, which is worse than the ideal model of simulation. Interestingly, the experiment shows that the Poisson ratio is smaller than the simulated Poisson ratio, which is due to the generation of convexity during the experiment process, resulting in a greater lateral displacement of the experimental model than that of the simulation model under the same strain, thus making the Poisson ratio relatively small.

## 6. Conclusions

In this paper, a modified 3D NPR lattice structure was presented based on the traditional concave structure and the star structure. Mechanical analyses of the models were undertaken at different angles through a quasi-static compression experiment and numerical simulation using Abaqus CAE commercial software. In addition, three control structures without star bodies were also designed. The fragmentation load–displacement curves were obtained via finite element simulations. Structural energy absorption was then obtained by integrating the load–displacement curves to obtain the mass-to-area ratio; the following conclusions can then be drawn:

1. The modified NPR structure designed here can effectively improve the stiffness of the structure and make up for the low stiffness of the negative Poisson relative to the metamaterial model;
2. Increasing the modified NPR structure of the star can effectively improve the stability of the structure and can avoid the phenomenon of “convexity” during destruction. The macroscopic stability of the structure is worse with increasing the ∅ angle of the star structure;
3. The energy absorption effect of the modified structure depends on the ∅ angle of the star structure rather than the concave angle $\emptyset_{2}$. The energy absorption effect of the modified NPR structure is the best when ∅ = 70.9°.

## Figures and Tables

**Table 1:** The three designed model plane parameters.

Type	A (mm)	B (mm)	∅ (°)	∅_2_ (°)	L (mm)
A	18.82	39.70	38.14	50	70
B	18.82	36.03	70.90	60	70
C	18.82	33.88	105.77	70	70

**Table 2:** Three parameters of the honeycomb structure.

Type	∅_2_	H (mm)	L_x_	L_z_	T (mm)
A	50	231.66	154.05	154.05	5
B	60	231.66	168.38	168.38	5
C	70	231.66	180.37	180.37	5

**Table 3:** Comparison of main parameters of SLM process and ordinary 316 L.

Classification	Elastic Modulus (GPa)	Yield Limit (MPa)	Tensile Strength (MPa)	$\mathrm{Density}\;(\mathrm{Kg}/m^{3})$	Poisson Ratio
SLM Specimen1	183.99	505	665	8.737	0.317
SLM Specimen2	197.51	500	665	8.791	0.316
SLM Specimen3	200.74	510	665	8.816	0.318
Ordinary 316 L	206	269.17	603.50	8.027	0.3

## Data Availability

This article details the data and results covered by this study.
